# Notes and Notices

**Published:** 1888-09

**Authors:** 


					﻿NOTES AND NOTICES.
Fob Sale.—“ Pathological Anatomy, Pathology, and Physical Diagnosis.”
A series of clinical reports, comprising the principal diseases, systematically
arranged; 100 full-page illustrations on 100 pages of text. By Prof. J. A.
Jeancon, M. D.; cost $25; for sale cheap.
				

